# Step-Adsorption of Vanadium (V) and Chromium (VI) in the Leaching Solution with Melamine

**DOI:** 10.1038/s41598-020-63359-z

**Published:** 2020-04-14

**Authors:** Hao Peng, Qian Shang, Ronghua Chen, Liuying Zhang, Ya Chen, Jing Guo

**Affiliations:** Chongqing Key Laboratory of Inorganic Special Functional Materials, College of Chemistry and Chemical Engineering, Yangtze Normal University, Fuling Chongqing, 408100 P. R. China

**Keywords:** Environmental chemistry, Engineering

## Abstract

The vanadium (V) and chromium (VI) was hard to separate directly due to the similar nature. In this paper, separation and recovery of vanadium (V) and chromium (VI) from a leaching solution was investigated by adsorption of vanadium (V) with melamine, followed by electro-reduction of chromium (VI) and adsorption of chromium (III) with melamine, respectively. The effects of experimental parameters including dosage of melamine, reaction temperature and reaction time on the adsorption process were investigated. The results showed that melamine was a good sorbent for adsorption of vanadium (V) and chromium (III). 99.89% of vanadium (V) was adsorbed by melamine at the optimal conditions, the adsorption kinetic was followed the pseudo-second-order model and the adsorption isotherm conformed to the Langmuir model. While the adsorption of chromium (III) was followed the pseudo-first-order model and the adsorption isotherm was conformed to the Freundlich model as the adsorption efficiency was 98.63% under optimal conditions.

## Introduction

Vanadium, chromium and their compounds are important national strategy resources and widely used in petrochemical, catalyst, iron steel industries, due to their excellent physicochemical properties^1–6^. In nature, vanadium and chromium are generally well-dispersed. To date, the main raw materials for vanadium recovery were converter vanadium slag^7–9^, stone coal^10–12^, steel slag^13^ and waste catalyst materials^14,15^. A considerable number of investigations had been performed to obtain optimum technology for recovery of vanadium and chromium. The common methods for leaching out vanadium and chromium were hydrometallurgical technologies, like sodium salt roasting-water leaching^16^, calcium roasting-acid leaching^17–19^, molten roasting and sub-molten technology^20,21^, and also oxidation leaching technologies^22,23^. Vanadium and chromium could be efficiently leached out through above technologies, but efficiently separated vanadium and chromium was hard to achieve as the high similarity physicochemical properties of vanadium and chromium.

Some experiments differed from the difference of the vanadium-chromium source had been conducted. A process consisted of acid leaching, selective oxidation hydrolysis precipitation were investigated to separate and recover chromium from vanadium-chromium bearing reducing slag^24^. The vanadium (III) and chromium (III) were leached out via acid leaching, and then vanadium (III) was oxidized to vanadium (V) with MnO_2_ while chromium was still existed as chromium (III). Then hydrolysis precipitation was used to separate vanadium (V) and chromium (III). In other research, hydrolysis and neutralization were used to separate and recover vanadium and chromium from vanadium (V)-containing chromate (VI) solution^25^. In this process, vanadium was hydrolyzed and the retained vanadium was removed with Fe (II) and Fe (III), then the chromium was reduced to chromium (III) and it was precipitated as Cr_2_O_3_·xH_2_O. Solvent extraction^12,26^ and ion exchange^27–29^ were also used to recover vanadium or chromium from the solution.

Melamine was usually used in manufacture of plastics, adhesives, cleaners, and yellow dye^30,31^, showed great adsorption activity when it was modified with metal-organic frameworks. Recent studies indicated that melamine derived nitrogen doped magnetic carbon demonstrated an excellent removal of Cr (VI)^32^, and the modified melamine-Cr-MOF had a 1.5-fold increase in adsorptive removal of artificial sweeteners from water^33^. Yin *et al*. found that melamine modified MOFs as absorbance had high adsorption capacity (205 mg g^−1^) and showed great adsorption performance on Pb (II)^34^. Though the introduction of melamine into MOFs was very meaningful, melamine as single absorbent had not been extensively studied by many researchers. In this paper, melamine was introduced as an efficient sorbent for vanadium (V) and chromium (VI) separation and recovery. The process was investigated by adsorption of vanadium (V) with melamine, followed by electro-reduction of chromium (VI) and adsorption of chromium (III) with melamine, respectively. The effects of experimental parameters including dosage of melamine, reaction temperature and reaction time on the adsorption process were investigated. Optimal adsorption conditions for vanadium (V) and chromium (III) were determined. Adsorption kinetic and thermodynamic were also investigated.

## Results and discussion

### Physicochemical characterization of melamine

**Figure 1:**
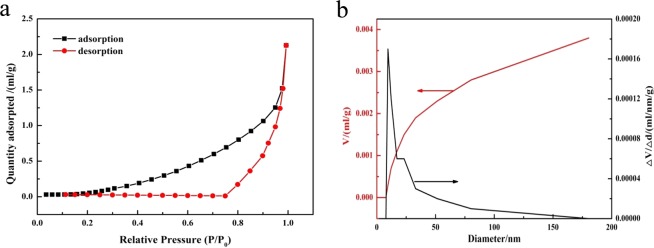
Physicochemical characterization of melamine. (**a**) N_2_ adsorption/desorption isotherms of the absorbance. (**b**) Pore size and pore volume distribution curves of melamine.

The specific surface area of melamine was measured on ASAP 2020 (Micrometrics, USA) at 77 K by determining the N_2_ adsorption/desorption isotherms. The result showed in Fig. 1a indicated that the isotherm of melamine was Type-II according to IUPAC classification^35^. When the relative pressure (P/P_0_) was less than or equal to 0.2, the adsorption and desorption curves of melamine were overlapped, which indicated that there were small micro pores existed in the surface of melamine and monolayer adsorption existed in the adsorption performance. The melamine showed a great specific surface area (8.71 m^2^/g) and adsorption pore volume (0.0040 mL/g). Figure 1b expressed the pore size and pore volume distribution curve of melamine. The peaks at 10 nm attributed to the aggregation of melamine particles, which indicated that the sorbent had narrow pore size distribution.

### Adsorption of vanadium

#### Effect of initial pH

**Figure 2:**
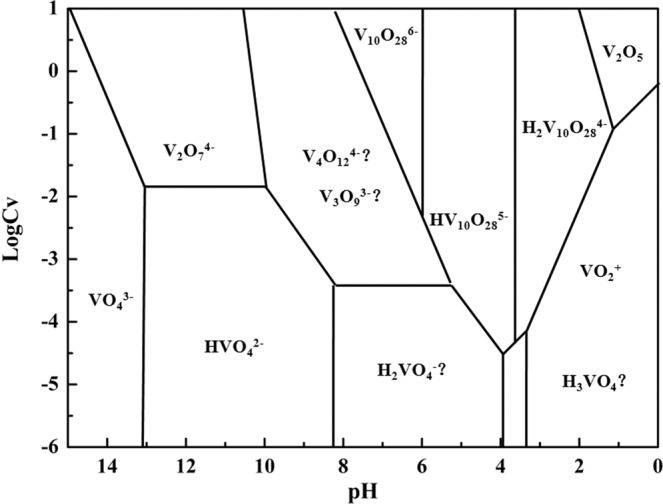
Relationship between the status of vanadium (V) in aqueous solution and the vanadium concentration and pH (25 °C)^36^.

The vanadium in aqueous solution had many species with the range of pH and concentration (showed in Fig. 2)^36^, and previous works showed that the adsorption process of vanadium with melamine was between VO_2_^+^ and melamine, which included chemical adsorption and physical adsorption^37,38^, Thus the initial pH value had significant effect on the adsorption process, the effect was investigated under the following conditions: reaction temperature of 90 λ, reaction time of 60 min, and mole ratio of n(melamine)/n(vanadium) = 1.0, respectively.

**Figure 3:**
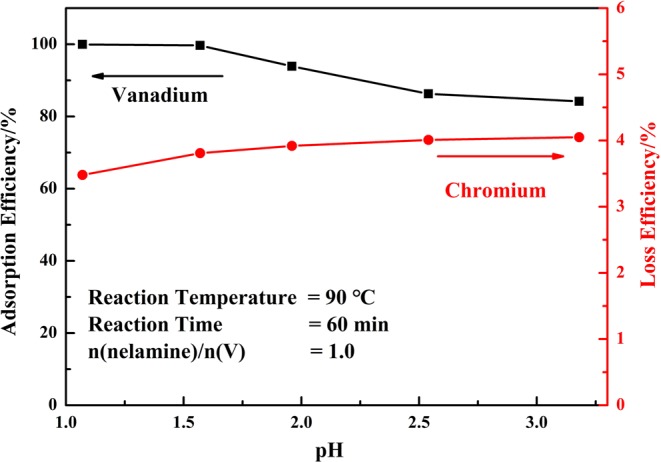
Effect of initial pH on the adsorption efficiency of vanadium (V) and loss efficiency of chromium (VI).

The results showed in Fig. 3 indicated that the adsorption efficiency of vanadium decreased as the pH increased. Previous works^34,39–41^ indicated that the melamine had good adsorption efficiency on metal ions, like Pb^2+^, Hg^2+^ and Ag^+^. The adsorption efficiency of vanadium was nearly 100% when the pH value was below 1.5. At this pH, the vanadium was existed as VO_2_^+^ and efficiently adsorbed by melamine. As the pH increased, the adsorption ability of melamine was decreased as the vanadium ions converted to polymer ions, like V_10_O_28_^6−^, HV_10_O_28_^5−^ or H_2_V_10_O_28_^4−^, which led to low adsorption efficiency. Thus, in order to obtain high adsorption efficiency of vanadium, the aqueous solution should be in acidic medium with low pH value.

The chromium (VI) in the solution existed as anion Cr_2_O_7_^2−^ in acidic medium and CrO_4_^2−^ in alkaline medium, which could not be absorbed by melamine. Some chromium ions were retained in the precipitate during the filtration process, which led to some loss of chromium. The results showed in Fig. 3 indicated that the loss efficiency of chromium (VI) was about 4.0% at various pH values, in other words, most chromium (VI) was still retained in the solution and it was efficiently separated with vanadium.

#### Effect of dosage of melamine

**Figure 4:**
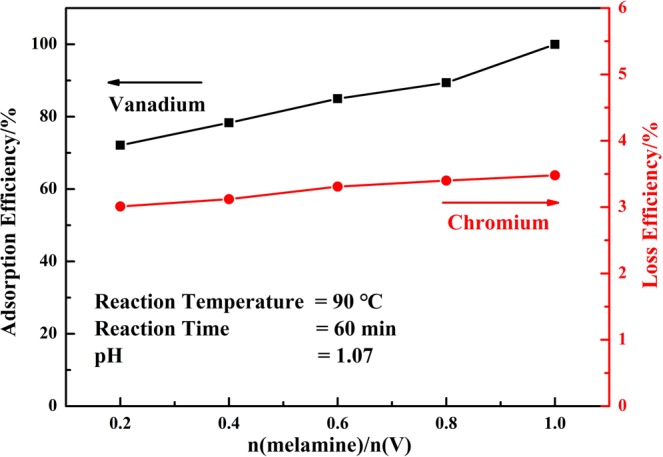
Effect of dosage of melamine on the adsorption efficiency of vanadium (V) and loss efficiency of chromium (VI).

The sorbent played an important role during the adsorption process, and the effect of the dosage of melamine on the reaction process was investigated under the following reaction conditions: reaction temperature of 90 °C, reaction time of 60 min, and initial pH value of the solution of 1.07, respectively. The results were shown in Fig. 4.

Melamine possessed three free amino groups and three aromatic nitrogen atoms in its molecule, and cationic form of vanadium (VO_2_^+^) was adsorbed on the surface of melamine and formed H-bond with amino groups^37,38^. More melamine could provide more active sites for vanadium ions for adsorption. The adsorption efficiency of vanadium was up to 99.89% at mole ratio of n(melamine)/n(V) = 1.0. While in the whole process, the loss efficiency of chromium (VI) was still below 4.0%.

#### Effect of reaction temperature

**Figure 5:**
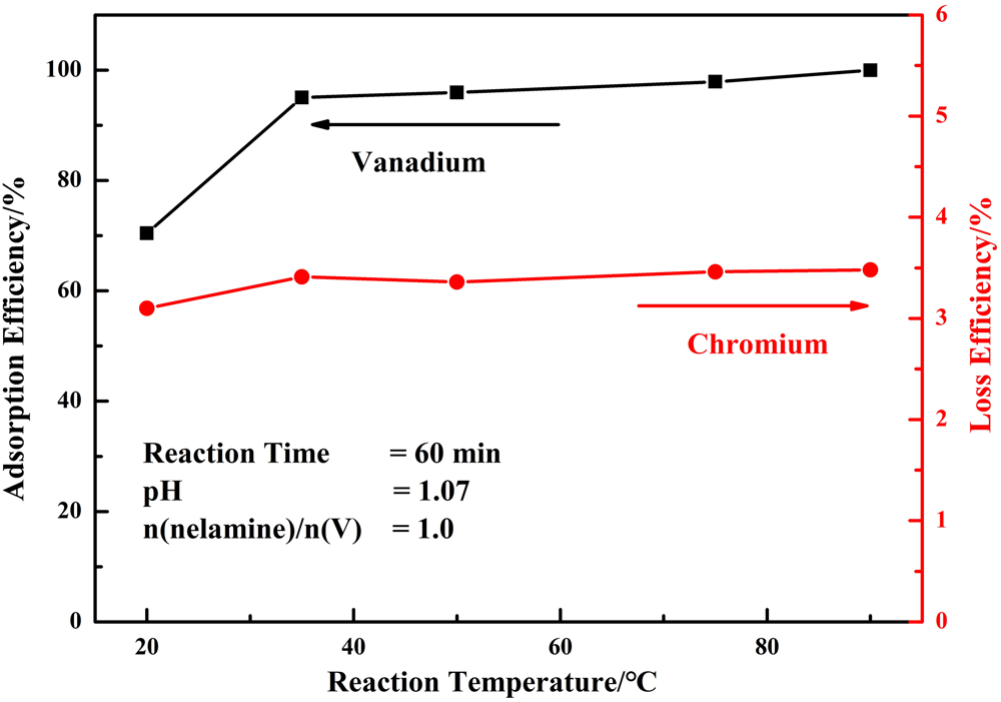
Effect of reaction temperature on the adsorption efficiency of vanadium (V) and loss efficiency of chromium (VI).

Reaction temperature was an important parameter during the adsorption process which affected the viscosity and diffusion resistance about the reaction medium. Figure 5 summarized the effect of reaction temperature on the reaction process. The results indicated that the adsorption efficiency of vanadium was affected significantly by the reaction temperature. The adsorption of vanadium was of about 70% at 20 °C and then increased to 95.02% as the reaction temperature increased to 35 °C, and also up to 99.89% at 90 °C. In other words, the adsorption process could be achieved efficiently at relatively low temperature for energy saving.

#### Effect of reaction time

**Figure 6:**
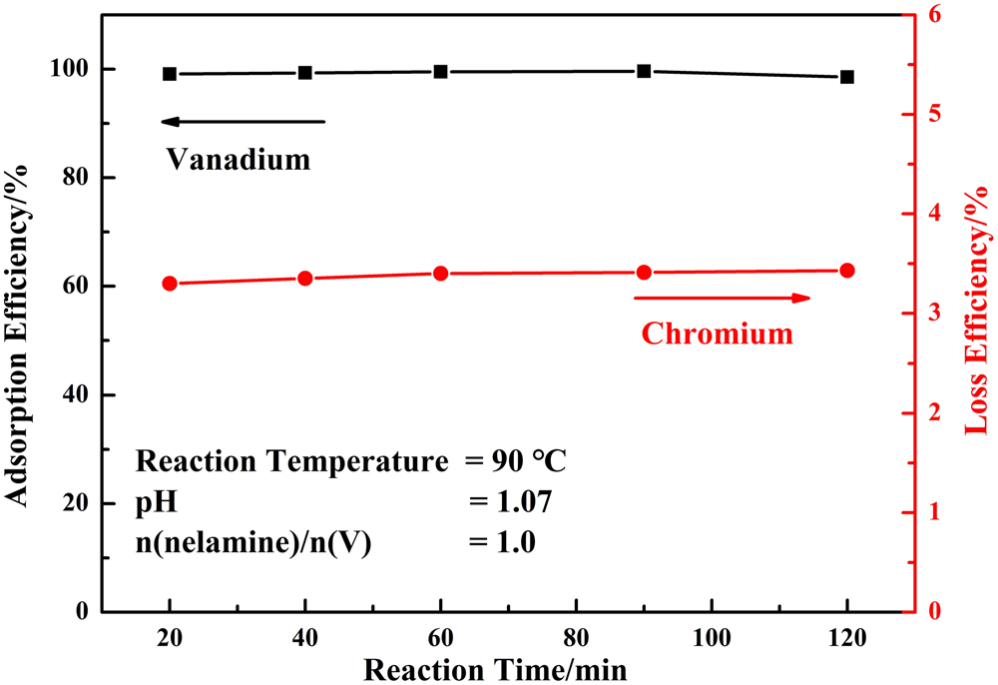
Effect of reaction time on the adsorption efficiency of vanadium (V) and loss efficiency of chromium (VI).

The effect of reaction time on the adsorption process was investigated as the other reaction conditions kept as constant: reaction temperature of 90 °C, dosage of melamine at mole ratio of n(melamine)/n(V) = 1.0, and initial pH value of the solution of 1.07, respectively. The results showed in Fig. 6 indicated that the melamine could adsorb enough vanadium in a relatively rapid rate. The adsorption efficiency of vanadium was up to 99.09% at its first 20 min and increased little at further reaction time. The adsorption process was reacted quickly and could achieved high adsorption efficiency at shortened reaction time.

### Electro-reduction of chromium

About 99.89% of vanadium was absorbed by melamine and most chromium (VI) was retained in the Filtrate I. The following step was the reduction of chromium (VI) to chromium (III) according to Fig. 12. Electro-reduction technology was applied to reduce chromium (VI) to chromium (III) and the detailed experiment conditions and results could be seen in ref. ^42,43^. After reduction, the chromium in the Filtrate I existed as chromium (III) and ready for further treatment.

### Adsorption of chromium

The chromium (III) was also treated by adsorption with melamine. The effects of experimental parameters including dosage of melamine, reaction temperature and reaction time on the adsorption efficiency of chromium were investigated.

#### Effect of dosage of melamine

**Figure 7:**
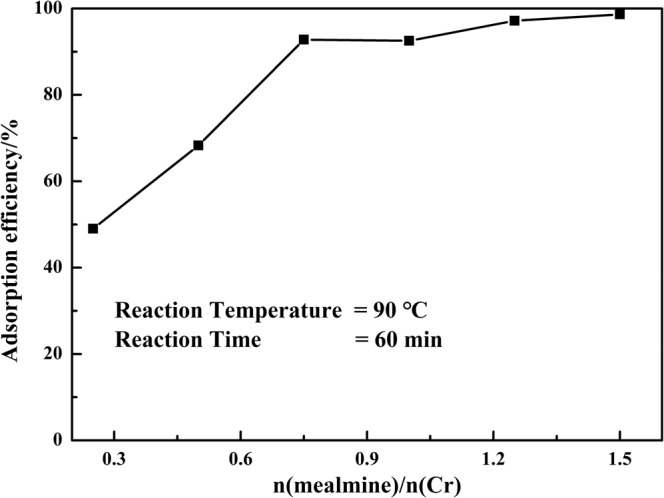
Effect of dosage of melamine on the adsorption efficiency of chromium.

Melamine as an sorbent was used to adsorb chromium (III) from the filtrate II. The dosage of melamine had significant effect on the adsorption efficiency of chromium (III) during the process. The effect of dosage of melamine on the adsorption efficiency of chromium (III) was investigated as other conditions kept as constant: reaction time of 60 min and reaction temperature of 90 °C. The results showed in Fig. 7 indicated that the adsorption efficiency of chromium (III) was increased with the increasing of dosage of melamine. The adsorption efficiency of chromium (III) firstly increased linearly, which increased from 49.00% at n(melamine)/n(Cr) = 0.25 to 92.81% at n(melamine)/n(Cr) = 0.75, and then increased slowly, up to 98.63% at n(melamine)/n(Cr) = 1.50.

#### Effect of reaction temperature

**Figure 8:**
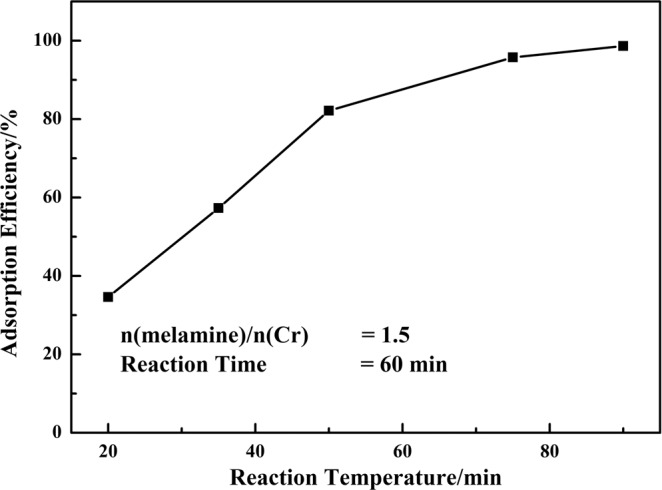
Effect of reaction temperature on the adsorption efficiency chromium.

The effect of reaction temperature on the adsorption efficiency of chromium was also investigated as the mole ratio of n(melamine)/n(Cr) = 1.5 and reaction time of 60 min. The results showed in Fig. 8 indicated that the adsorption efficiency of chromium (III) increased with the increasing of reaction temperature. High temperature was favorable for the adsorption process as the adsorption process of chromium (III) was endothermic. In other words, the diffusion rate of chromium (III) ions increased and the viscosity of the solution decreased along with the increase of reaction temperature, which was beneficial for the contact of chromium (III) and melamine, and contributing to adsorption of chromium (III). The adsorption efficiency of chromium (III) was up to 98.63% at reaction temperature of 90 °C.

#### Effect of reaction time

**Figure 9:**
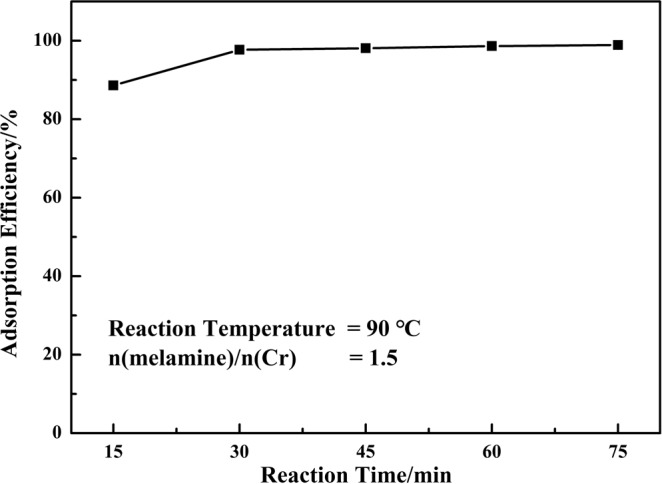
Effect of reaction time on the adsorption efficiency of chromium.

Figure 9 showed the effect of reaction time on the adsorption efficiency of chromium (III). At the beginning of the adsorption process, the vacant sites on the surface of melamine was enough, the chromium (III) ions could easily react with the active sites and been adsorbed. With the increase of reaction time, the adsorption sites of melamine would reach its saturation, the adsorption rate decreased, and the adsorption efficiency increased slowly. The adsorption efficiency of chromium (III) was up to 88.60% within 15 min, which indicated that the melamine was an effective absorbent for adsorption of chromium (III).

### Adsorption kinetics

In order to understand the adsorption process, pseudo-first-order and pseudo-second-order kinetic models were applied to analyze the experimental data. These two kinetic models could be expressed as Eqs. (1) and (2)^44^, respectively.

1 $$\mathrm{ln}({\rm{Qe}}-{\rm{Qt}})=\,\mathrm{ln}({\rm{Qe}})-{{\rm{k}}}_{1}{\rm{t}}$$

2 $$\frac{{\rm{t}}}{{\rm{Qt}}}=\frac{1}{{{\rm{k}}}_{2}{{\rm{Qe}}}^{2}}+\frac{1}{{\rm{Qe}}}{\rm{t}}$$

Where, *Qe* is the adsorption capacity at equilibrium, mg/g; *Qt* is the adsorption capacity at time t, mg/g. And *k*_1_ is the rate constant of pseudo-first-order, /min; and *k*_2_ is the pseudo-second-order sorption model rate constant, g/(mg·min).

**Figure 10:**
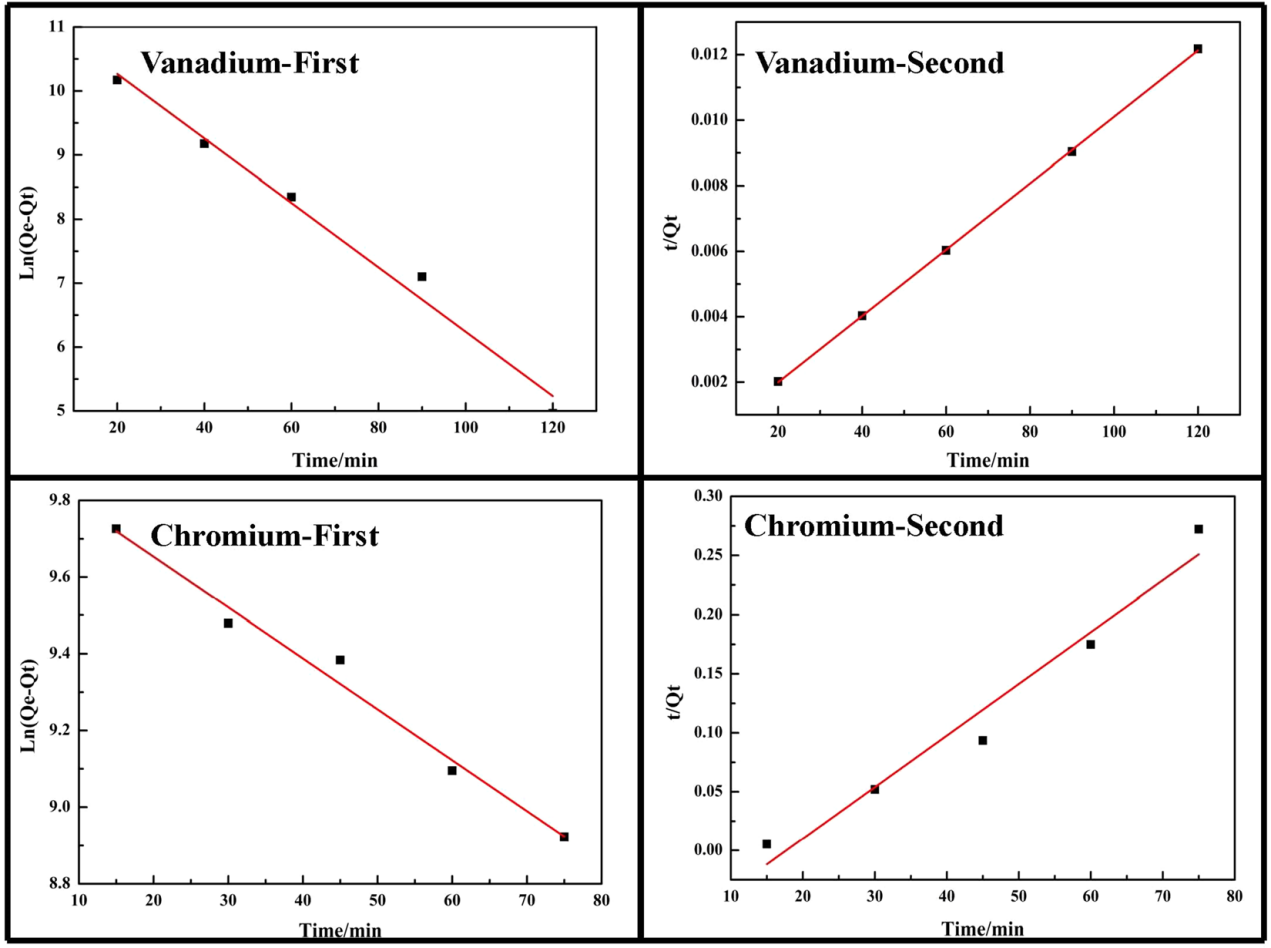
Adsorption kinetic models for vanadium (V) and chromium (III) with melamine (First-Pseudo-first-order model and Second-Pseudo-second-order model).

The correlation coefficients (*R*^2^), *k*_1_*, k*_2_ and *Qe* values were all shown in Table 1, and the linear plots of these two models were shown in Fig. 10. Taking vanadium adsorption process for consideration, the *Qe* of the pseudo-first-order model was 59874 mg/g, while the *Qe* of the pseudo-second-order model was 9865 mg/g, which was close to the experimental results discussed above (the experimental data was 9998 mg/g for adsorption efficiency of 99.89%). And the *R*^2^ of the pseudo-second-order model was 0.9998. It was suggested that the vanadium adsorption onto the melamine was followed the pseudo-second-order kinetic model.

**Table 1:** Constants and correlation coefficients of pseudo-first order and pseudo-second order kinetic models for adsorption of vanadium (V) and chromium (III) with melamine.

	Pseudo-first-model			Pseudo-second-model		
	*Qe (mg/g)*	*k*_1_ *(min*^*−*1^)	*R*^2^	*Qe (mg/g)*	*k*_2_ *(g/mg/min)*	*R*^2^
Vanadium	59874	0.05033	0.9815	9865	2.99 exp (12)	0.9998
Chromium	19930	0.01328	0.9788	228.3	6.76 exp (5)	0.9543

For consideration of chromium, the *Qe* was 19930 mg/g for pseudo-first-order model, which was close to the experimental results discussed above (the experimental data was 19602 mg/g for adsorption efficiency of 98.63%). It was suggested that the chromium adsorption onto the melamine was followed the pseudo-first-order kinetic model.

### Adsorption isotherms

Equilibrium adsorption isotherm could provide information about the surface property of sorbent and the adsorption behavior and it was important for the design of adsorption systems^45^. In this study, Langmuir isotherm models and Freundlich’s isotherm model expressed as Eqs. (3) and (4) were applied to analyze the experimental data^46^.

Langmuir equation:

3 $$\frac{{\rm{Ce}}}{{\rm{Qe}}}=\frac{1}{{{\rm{Q}}}_{0}{{\rm{K}}}_{{\rm{L}}}}+\frac{{\rm{Ce}}}{{{\rm{Q}}}_{0}}$$

Where, *K*_*L*_ is the adsorption equilibrium constant, L/mg; *Q*_0_ is the maximum monolayer adsorption capacity, mg/g; and *Qe* is the amount adsorbed on a unit mass of the absorbance, mg/g.

Freundlich equation:

4 $${\rm{lnQe}}={{\rm{lnK}}}_{{\rm{f}}}+\frac{1}{n}{\rm{lnCe}}$$

Where, *K*_*F*_ is the Freundlich constants related to sorbent capacity, (mg/g) (L/g)^n^; *1/n* is Freundlich constants related to adsorption intensity of absorbance. The value of *n* from Freundlich model represented the adsorption characteristic. *n* = 2–10 predicted good adsorption, and n = 1–2 showed moderately difficult adsorption ability, and n < 1 found a poor adsorption characteristic.

**Figure 11:**
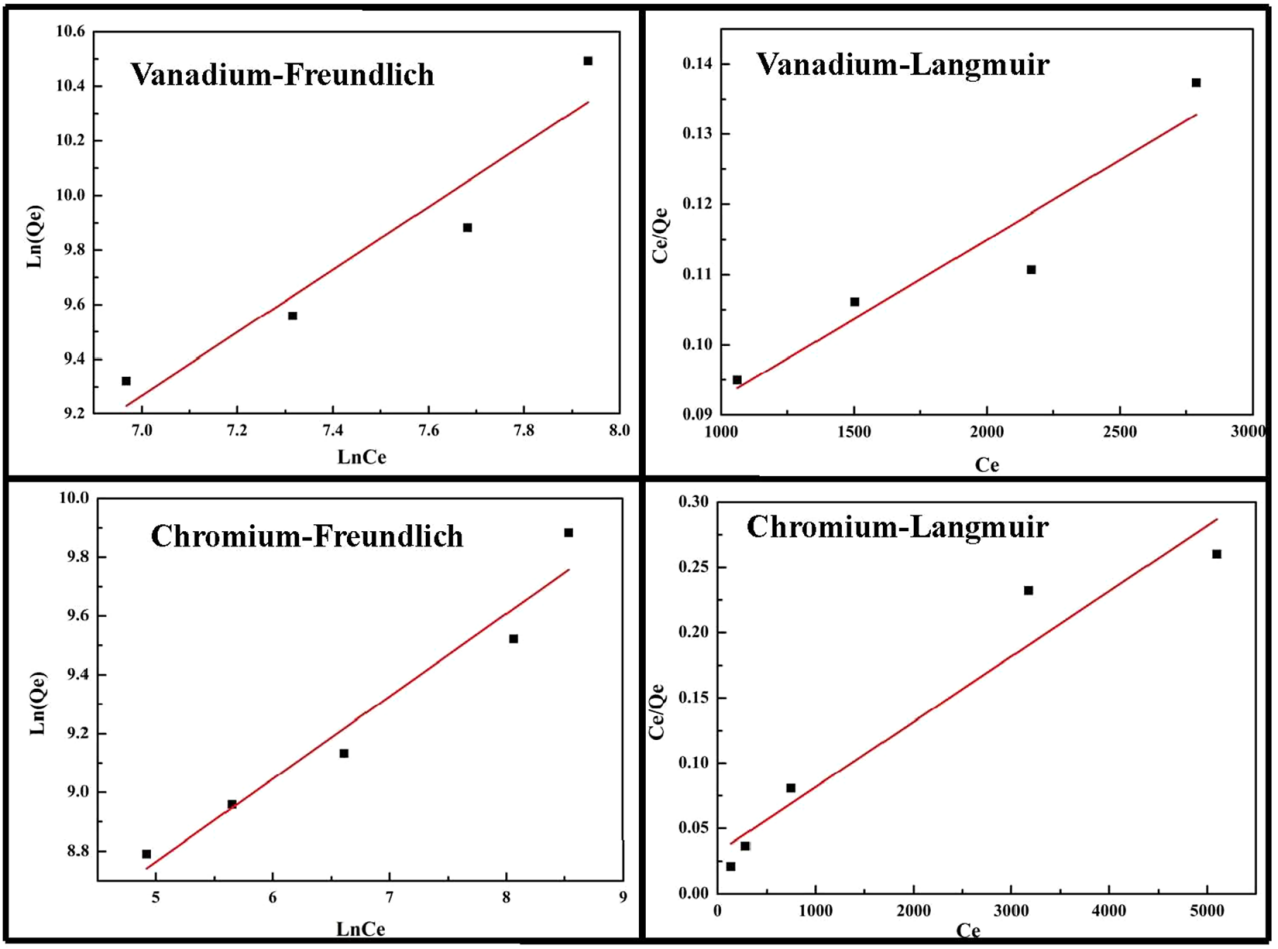
Adsorption isotherms for the adsorption of vanadium (V) and chromium (III).

Figure 11 showed the Freundlich isotherms and Langmuir isotherms for vanadium (V) and chromium (III) adsorption onto the melamine at 90 °C, respectively, and the results of relative parameters (*K*_*L*_*, Q*_*0*_, *K*_*F*_ *and n*) were shown in Table 2. The *n* of vanadium and chromium (III) adsorption onto the melamine was 0.87 and 3.56, respectively, which indicated that the Freundlich isotherm was not suitable for adsorption of vanadium. The monolayer adsorption capacity of melamine for vanadium adsorption calculated from the Langmuir isotherm was 44326 mg/g, the correlation coefficients *R*^2^ of Langmuir model was lower than Freundlich model for adsorption of chromium (III), which indicated that the adsorption of chromium (III) onto the melamine conformed to the Freundlich model.

**Table 2:** Parameters of adsorption isotherms of vanadium (V) and chromium (III) with melamine.

	Langmuir isotherm			Freundlich isotherm		
	Q_0_ (mg/g)	K_L_ (L/mg)	R^2^	K_F_ (L/mg)	n	R^2^
Vanadium	44326	3097	0.8567	3.4041	0.87	0.8736
Chromium	19972	630.7	0.9205	1556	3.56	0.9387

### Thermodynamic analysis

Gibbs free energy change (△G^0^) could provide an insight into the adsorption mechanism and behavior^47^. The △G^0^ value was calculated to −24.26 kJ/mol and −19.45 kJ/mol for vanadium and chromium at 363 K, respectively, it was indicated that the adsorption behavior of vanadium (V) and chromium (III) onto the melamine was thermodynamically favorable and spontaneous^48^.

## Conclusions

The vanadium (V) and chromium (VI) in the leaching solution was separated efficiently by step-adsorption with melamine. The following conclusions could be deduced:

(1) Melamine was a good absorbent for adsorption of vanadium and chromium (III). The adsorption efficiency of vanadium was up to 99.89% at optimal conditions: initial pH value of 1.07, reaction temperature of 90 °C, reaction time of 60 min, and mole ratio of n(melamine)/n(vanadium) = 1.0. The adsorption process of vanadium was well described by the pseudo-second-order model and the adsorption isotherm was conformed to the Langmuir model.

(2) The chromium (VI) was easily reduced to chromium (III) with the electro-reduction technology, and the chromium (III) was efficiently adsorbed with melamine. The adsorption efficiency of chromium (III) was achieved at 98.63% at reaction temperature of 90 °C, reaction time of 60 min, and mole ratio of n(melamine)/n(chromium) = 1.5. And the adsorption kinetic followed the pseudo-first-order model and the adsorption isotherm was conformed to the Freundlich model.

## Materials and methods

### Materials

The leaching solution contained 7.6 g/L vanadium and 30 g/L chromium was obtained by oxidative-alkaline leaching with H2O2 of vanadium-chromium reducing residue under following reaction conditions: the liquid to solid ratio of 4.0 ml/g, residue particle size of <200 mesh, the mass ratio of NaOH-to-residue of 1.0 g/g, the volume ratio of H2O2-to-residue of 1.2 ml/g, reaction temperature of 90 °C and reaction time of 120 min^49,50^. All reagents including melamine (C_3_N_6_H_6_), sulfuric acid (H_2_SO_4_), and phosphate acid (H_3_PO_4_) were analytical grade and all solution was prepared with deionized water with a resistivity greater than 18 MΩ/cm (HMC-WS10).

### Experimental procedure

#### Adsorption of vanadium

**Figure 12:**
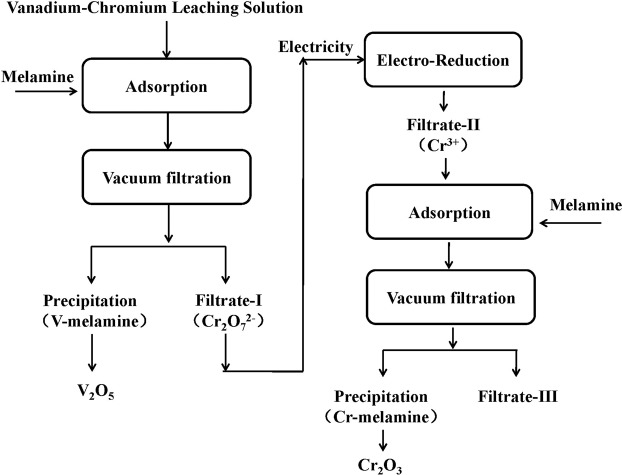
Flow sheet of separation and recovery of vanadium and chromium.

The experiments were performed according to the flow sheet as shown in Fig. 12, which included the procedures of adsorption of vanadium with melamine, electro-reduction of chromium (VI) and adsorption of chromium (III) with melamine.

The adsorption performance of vanadium was performed in a 200 mL beaker with a thermostatic mixing water bath pot at a shaking speed of 500 rpm. A predetermined amount of vanadium-chromium leaching solution was added to the beaker and then heated to a predetermined temperature. Next, a predetermined amount of melamine was added to the beaker. After the required reaction time, the Filtrate-I was separated from the precipitation by vacuum filtration. The concentration of vanadium and chromium in the filtrate was determined by inductive couple plasma-optical mission spectrometry (ICP-3000).

The leaching efficiency of vanadium (η_1_) and loss efficiency of chromium (η_2_) was calculated in Eqs. (5) and 6), as followed:

5 $${\eta }_{1}=\frac{{{\rm{V}}}_{1}\cdot {{\rm{C}}}_{1}-{{\rm{V}}}_{2}{{\rm{C}}}_{2}}{{{\rm{V}}}_{1}\cdot {{\rm{C}}}_{1}}\times 100 \% $$

6 $${\eta }_{2}=\frac{{{\rm{V}}}_{1}\cdot {{\rm{C}}}_{3}-{{\rm{V}}}_{2}{{\rm{C}}}_{4}}{{{\rm{V}}}_{1}\cdot {{\rm{C}}}_{3}}\times 100 \% $$

Where, *C*_1_*, C*_2_, is the concentration of vanadium in the vanadium-chromium leaching solution and Filtrate-I, g/L; *C*_3_*, C*_4_, is the concentration of chromium in the vanadium-chromium leaching solution and Filtrate-I, g/L; *V*_1_*, V*_2_, is the volume of vanadium-chromium leaching solution and the Filtrate-I, L.

#### Electro-reduction of chromium (VI)

The electro-reduction of chromium (VI) from the Filtrate-I was also performed in a 200 mL beaker with a thermostatic mixing water bath pot at a shaking speed of 500 rpm. The chromium (VI) was reduced to chromium (III) with electricity at optimal conditions^42,43^. The filtrate-II contained chromium (III) was obtained.

#### Adsorption of chromium (III)

The adsorption of chromium (III) from the filtrate-II was also performed in a 200 mL beaker with a thermostatic mixing water bath pot at a shaking speed of 500 rpm. A predetermined amount of filtrate-II was added to the beaker and then heated to a predetermined temperature. Next, a predetermined amount of melamine was added to the beaker. After the required reaction time, the filtrate-III was separated from the precipitation by vacuum filtration. The concentration of chromium (III) in the filtrate-III was determined by inductive couple plasma-optical mission spectrometry (ICP-3000).

The adsorption efficiency of chromium (η_3_) was calculated in Eq. (7), as followed:

7 $${\eta }_{3}=\frac{{{\rm{V}}}_{3}\cdot {{\rm{C}}}_{5}-{{\rm{V}}}_{4}{{\rm{C}}}_{6}}{{{\rm{V}}}_{3}\cdot {{\rm{C}}}_{5}}\times 100 \% $$

Where, *C*_5_*, C*_6_, is the concentration of chromium (III) in the Filtrate-II and Filtrate-III, g/L; *V*_3_*, V*_24_, is the volume of Filtrate-II and Filtrate-III, L.
